# A comparative effectiveness study of two culturally competent models of diabetes self-management programming for Latinos from low-income households

**DOI:** 10.1186/s12902-017-0192-4

**Published:** 2017-07-24

**Authors:** Janet Page-Reeves, Lidia Regino, Cristina Murray-Krezan, Molly Bleecker, Erik Erhardt, Mark Burge, Elaine Bearer, Shiraz Mishra

**Affiliations:** University of New Mexico Health Sciences Center, Albuquerque, USA

**Keywords:** Diabetes, Self-care, Cultural competency, Hispanic Americans, Low-income

## Abstract

**Background:**

Diabetes risk is extremely high for Latinos from low-income households. Health guidelines recommend that individuals learn strategies to self-manage their diabetes, but getting people to adopt required lifestyle changes is challenging and many people are not able to prevent their pre-diabetes from escalating or effectively control their diabetes. Systematic reviews show that culturally competent self-management programs can significantly improve diabetes outcomes and different models for culturally competent programming have been developed.

**Methods:**

This patient-engaged study will compare the effectiveness of two distinct evidence-based models for culturally competent diabetes health promotion at two sites that serve a large Latino patient population from low-income households: *1) The Diabetes Self-Management Support Empowerment Model*, an educational session approach*,* and *2) The Chronic Care Model,* a holistic community-based program*.* Data collection will involve interviews, focus groups, surveys and assessments of each program; and testing of patient participants for A1c, depression, Body Mass Index (BMI), and chronic stress with hair cortisol levels. We will recruit a total of 240 patient-social support pairs: Patients will be adults (men and women over the age of 18) who: *1.)* Enter one of the two diabetes programs during the study; *2.)* Self-identify as “Latino;” *3.)* Are able to identify a social support person or key member of their social network who also agrees to participate with them; *4.)* Are not pregnant (participants who become pregnant during the study will be excluded); and *5.)* Have household income 250% of the Federal Poverty Level (FPL) or below. Social supports will be adults who are identified by the patient participants. **PRIMARY OUTCOME:** Improved capacity for diabetes self-management measured through improvements in diabetes knowledge and diabetes-related patient activation. **SECONDARY OUTCOME**: Successful diabetes self-management as measured by improvements in A1c, depression scale scores, BMI, and circulating levels of cortisol to determine chronic stress.

**Discussion:**

Our hypothesis is that the program model that interfaces most synergistically with patients’ culture and everyday life circumstances will have the best diabetes health outcomes.

**Trial registration:**

This study was registered with ClinicalTrials.gov on December 16, 2016 (Registration #NCT03004664).

## Background

Although diabetes is a national health crisis, risk is not the same for everybody. Individuals from minority and ethnic populations and those with low-income status are at significantly higher risk [1]. Health guidelines emphasize the importance of patient self-care over narrow reliance on medical treatments for reducing the health impact of diabetes and improving diabetes health outcomes. However, systematic reviews have repeatedly demonstrated that culturally competent health promotion approaches that account for culture and the social context of poverty can be key to improving health outcomes [2–11]. In particular, culturally competent self-management interventions have been shown to significantly improve both glycemic control and behaviors related to diet and physical activity, and also to increase diabetes-related knowledge. A variety of different models have been developed to create “culturally competent” diabetes self-management programs [2–11]. Yet, there is no agreement on what cultural competence specifically means or entails, and, because of a continued emphasis on individual behavior in approaches to diabetes health promotion, the design of self-management programs does not always create cultural competence in a way that makes sense in relation to patients’ lives or improve their health.

## Methods/Design

**Fig. 1:**
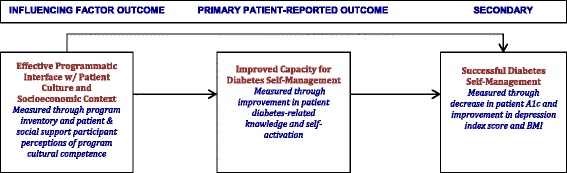
Conceptual model

This patient-engaged project compares the effectiveness of two distinct evidence-based models for culturally competent diabetes self-management health promotion currently being implemented by programs available to Latino patients from low-income households in Albuquerque, New Mexico. We hypothesize that the program model that interfaces most synergistically with patient’s culture and everyday life circumstances will have the better diabetes health outcomes (See Fig. 1: Conceptual Model).

### Aims

*Aim #1* *: Characterize the ways that two distinct culturally competent diabetes self-management programs* **interface** *with patient culture and socioeconomic context.*

*Aim #2* *: Measure and compare improvement in patient* **capacity** *for diabetes self-management.*

*Aim* *#3* *: Measure and compare patient* **success** *at diabetes self-management.*

### Design

Data will be gathered at baseline, three, six and 12 months by community data collectors who will be members of the research team. Each of the comparator sites is implementing a program in a “real life” setting, thus providing the opportunity for a pragmatic assessment of the comparative effectiveness of the program models under externally valid and generalizable conditions.

### Study population demographics

New Mexico, one of four majority minority states, has the largest percentage of Latinos in the United States (46.3% of the state’s population) [12]. Of New Mexico’s nearly one million Latino residents, most are of Mexican ancestry (62%) [13]. In Albuquerque, 47% of the population is Latino [14]. In New Mexico, 18.9% of Latinos live in households below the poverty level and 12.4% of Latinos have diabetes [15]. Census data reveal that Mexican-heritage Latinos tend to self-identify as racially “white” (53%) or as “some other race” (37%) [16]. This fact and the specific racial make-up of the New Mexico population influence our anticipated enrollment.

Source of Recruitment, Including the Volume of Eligible Patients Available and Expected Participation Rate. We will recruit 240 patient participants from the two programs through our site partner institutions. Estimates of current clinical use suggest that we will be able to successfully recruit participants to meet our target enrollment (See Table 1).

**Table 1:** Target Enrollment

Program	# Patients (annually)	# (%) latino	# (%) at or below 250 % FPL	# with diabetes	Annual target enrolment	Total target enrolment
CCM	1032	981 (95%)	1032 (100%)	142	40 Patients and 40 Social supports	120 Patients and 120 Social supports
DSMS	427	145 (34%)	149 (35%)	427	40 Patients and 40 Social supports	120 Patients and 120 Social supports

*CCM* chronic care model

*DSMS* the diabetes self-management support empowerment model

*FPL* Federal poverty level

*Comparator #1. The Diabetes Self-Management Support Empowerment Model (DSMS)* [17]. The DSMS is a patient-centered, theoretically based educational framework. It combines a series of clinically informed group didactic sessions that use a patient self-determination approach to empower patients to take control of their own diabetes health with follow-up supports to sustain self-management gains achieved during the sessions. The DSMS requires that educators acquire proficiency in culturally competent supportive care across the lifespan as one of five domains for certification. Educators are expected to be informed about, anticipate, and be aware of specific challenges that might accrue in the patient’s diabetes self-management experience.

*Comparator #2. The Chronic Care Model (CCM)* [11, 18]*.* The CCM involves six synergistic domains: *1.)* Improved access to care, *2.)* Patient self-management support, *3.)* Patient decision support, *4.)* Care coordination, *5.)* Integrated health information systems, and *6.)* Access to community resources. To create a holistic care regime, the CCM focuses on addressing social determinants of health by meeting the medical, cultural, and linguistic needs of patients through integration of cultural norms and social relationships from the patient population into the program design.

### Participant characteristics

This project will enroll three types of participants for different components of the study:

- Patients
- Social supports
- Program staff and providers

#### Patient (*n* = 240) & social support (*n* = 240) participants

Participants for this study will be enrolled as patient-social support dyads. One hundred twenty patients and their corresponding social supports will be enrolled at each site. All patient participants will be adults (men and women over age 18) who: *1.)* have been identified by a provider as having pre-diabetes (A1c between 5.7–6.4%) or diabetes (A1c ≥6.5%) and enter one of the two diabetes programs during the study; *2.)* self-identify as “Latino” or “Hispanic;” *3.)* are able to identify a social support person or key member of their social network who also agrees to participate with them; *4.)* are not pregnant (participants who become pregnant during the study will be excluded); and *5.)* have household income 250% of the FPL or below. Social support participants will be adult individuals who are identified by the patient participants.

#### Subset of patient and social support participants for interviews (72) and focus groups (72)

At each of the sites, we will identify a purposive sample of interviewees and focus group participants from those already recruited to be in the study and invite them to participate.

#### Staff & providers (*n* = 36)

As part of our program assessment, we will identify up to 6 key program staff and/or providers per site each year.

### Description of processes

We will use a variety of data sources to compare the effectiveness of the two models including program inventories, surveys, interviews, focus groups, and patient physical measures (A1c, BMI, PHQ-9, and hair cortisol). Domains of inquiry include: *a.)* program accommodation of patients’ language preference, cultural values, and socio-economic limitations, *b.)* program-related interpersonal interaction and communication, *c.)* program design to encourage social support among participants, *d.)* social “fit” with and social support from program peers, *e.)* social support from program staff, *f.)* diabetes health knowledge, *g.)* diabetes self-activation, *h.)* stress management, *i.)* A1c control, and *j.)* lifestyle changes to support diabetes health. Program assessment data will allow us to characterize the nature of each program and its approach to cultural competence. Survey responses and clinical test results from a large sample at each site will yield empirical (quantitative) data on patient-reported outcomes. Interviews and focus groups with a subset of participants will provide rich, in-depth, contextually meaningful (qualitative) data regarding the domains of inquiry from the perspective of patients, social supports, and program staff/providers.

All study activities will be conducted in English or Spanish, depending on the language preference of the participant. For each component of the study, the participant will receive a $50 merchandise card.

#### Programmatic assessment

We will inventory each program regarding program design, size, structure, operation, and theoretical/philosophical orientation; professional qualifications/training of program providers; activities or resources available through the programs; strategies in place for Spanish language use or acceptance, inclusion of social supports and family, accommodation of challenges created by patients’ limited socioeconomic circumstances, and the inclusion of stress management techniques; and data on referrals to the program, sign-ups, participation, no-shows, and attrition. To gather qualitative information, we will conduct interviews with providers and staff at each site. We will assess patient and social support participants’ perceptions of the program interface using a cultural competence survey [19, 20] that asks about physician bias, inter-cultural understanding, respectful interactions, language barriers, experiences of discrimination, and issues of trust. To gather qualitative information about cultural competence, we will include questions on programmatic interface in interviews and focus groups with patients and social supports (described below).

#### Surveys

We will hire four individuals from the population of study to work as Data Collectors. The Data Collectors will administer the survey orally to all patient and social support participants at baseline (when they enter the study), with follow-up at three, six, and 12 months. All participants will be asked to provide: *1.)* Contact information to be used for follow-up, notification of high A1c results, and to invite the participant to the end-of-project presentation of data to study participants; and *2.)* Demographic information to be used to characterize the participant and to be used as covariates in the proposed analyses. The survey will consist of questions from or modified from four tools validated and found to be reliable in our population:

*1.)* The *Consumer Assessment of Healthcare Providers and Systems Cultural Competence Set* (CAHPS-CC) [19, 20] [used as part of the programmatic assessment discussed in the preceding paragraph—will be administered at 3, 6 and 12 months only].

2*.)* The *Diabetes Knowledge Questionnaire* (DKQ) [21–23].

*3.)* The *Patient Activation Measure* (PAM-13) [24–32].

*4.)* The *Patient Health Questionnaire 9* (PHQ-9) [33–36].

#### Interviews

We will conduct 72 interviews with patients and social supports (12 per year × 2 sites × 3 years). We believe that data from 12 interviews per site per year will capture a sufficient range of responses to achieve thematic saturation [37]. Interviews will last one to 2 h and will be semi-structured. We will audio-record interview sessions and recordings will be transcribed and translated if necessary.

#### Focus groups

We will conduct 12 focus groups (2 per year × 2 sites × 3 years). Focus group participants will be distinct from those recruited to participate in the interviews described in the preceding paragraph. Focus groups will include six distinct participants each. Focus groups will last one to 2 h and will follow a semi-structured focus group guide. We will audio-record focus group sessions and recordings will be transcribed and translated if necessary.

#### Physical measures

We will obtain the following measures from patients at baseline, 3, 6, and 12 months:

##### A1c

The phlebotomy-trained Data Collector will draw blood samples at the same time they administer the survey. A1c will be tested on whole blood using the DCA Vantage 2000 analyzer.

##### Body mass index (BMI)

Patient participants’ BMI will be calculated using height and weight measurements obtained by the data collectors. Height and weight measurements will be taken using a standardized protocol. Two measurements will be taken for height and two for weight for each participant at each data collection point. An average of the two measures of height and an average of the two measures of weight will be used in the BMI calculation. The calculation will be done automatically in the database.

##### Hair cortisol

To obtain objective information about the biological level of chronic stress, we will measure cortisol in hair. Hair cortisol is emerging as a reliable test for circulating levels of cortisol, which is an indication of chronic stress [38–40]. Hair will be gathered from patients only and not from social supports, and will only be gathered at two time points: baseline and 6 months (note: hair samples will only be gathered at baseline and 6 months).

### Database

The data just described will be managed using REDCap software [41] which includes data capture, storage, and reporting capabilities. Surveys will be designed in REDCap to capture study participants’ contact information, demographic information, and responses to the survey questions. The physical measures of patient participants will also be stored in the REDCap database, and recruitment, data collection appointments, and participation in qualitative data gathering activities will also be tracked using the software.

### Statistical analysis

#### Hypothesized effect sizes and sample size requirements for outcomes

We hypothesize that the CCM model will be superior to the DSMS in its ability to increase diabetes knowledge and patient activation, lower A1c, and improve depression scale scores, BMI, and levels of stress among participants. Our hypothesized effect sizes and measures of variability are based on previously reported improvements as measured by the various instruments (cited for each measure in Table 2).

**Table 2:** Hypothesized effect sizes and measures of variability

Outcomes	Measures	Method/Instrument	Hypothesis^a^	Hypothesized Change score, Δ_DSCS-CCM_ (SD)	Hypothesized Cohen’s *f* effect size	Power
Primary Outcome: Improved Capacity for Diabetes Self-Management	Diabetes Knowledge	DKQ summed score	The CCM Model will result in a larger increase in DKQ summed scores	2.2 (3.8) (references [21–23])	0.09	96%
	Patient Activation	PAM-13 raw score	The CCM model will result in a larger increase in PAM-13 raw scores	12.7 (24.8) (references [24–32, 44])	0.07	85%
Secondary Outcome: Successful Diabetes Self-Management	A1c	% A1c	The CCM model will result in a larger decrease in percent A1c	−0.5 (1.0) (references [45–48])	0.06	84%^b^
	BMI	BMI	The CCM model will result in a larger decrease in BMI	−1.5 (3) kg/m^2^ (references [47, 49])	0.06	84%^b^
	Depression	PHQ-9 summed score	The CCM model will result in a larger decrease in PHQ-9 summed scores	−3 (6) (references [50–52])	0.06	84%^b^
Exploratory Outcome: Improved Stress Management	Hair Cortisol	Cortisol amount (pg/mg)	The CCM model will result in greater decrease in average cortisol levels	4 (6) (references [38–40, 53–58])	0.05	95%

^a^Changes over time are from baseline to 6 months

^b^It is coincidental that the power (and effect sizes) are the same for each of the three secondary outcomes

*BMI* body mass index

*DKQ* diabetes knowledge questionnaire

*PAM-13* patient activation measure 13-item instrument

*PHQ-9* patient health questionnaire 9-item instrument

*Pg/mg* picogram of cortisol per milligram of hair

#### Sample size and power calculations

We will recruit *N* = 240 patient-social support pairs (*n* = 120 per site) with anticipated 20% attrition to obtain complete data on at least *n* = 96 pairs per site. Table 2 describes power for hypothesized effect sizes between the CCM and DSMS between the change scores from baseline to 6 months on the DKQ, and PAM-13. Similarly, we report power for the secondary measures (A1c, BMI, and PHQ-9), as well. We assumed a two-sided type I error rate and adjusted for the number of comparisons made (two comparisons for the co-primary outcomes; three for the co-secondary outcomes) using a Bonferroni correction (α = 0.025, α = 0.017, respectively). The power analyses for detecting site differences among change scores were based on multiple linear regression models including demographic characteristics, participants’ perceived cultural competence of providers (CAHPS-CC), and social supports’ change scores on the DKQ, PAM-13, and PHQ-9 as covariates. We report Cohen’s *f* effect sizes based on the regression method [42, 43].

#### Statistical analysis

We will use descriptive statistics to summarize patient and social support characteristics. We will calculate means and standard deviations or medians and quartiles for continuous variables and will compare them across sites by *t* test or Wilcoxon rank sum test, depending on the distribution of the data. We will calculate frequencies and percentages for categorical variables and will compare them with the chi-square test or Fisher’s exact test, as appropriate. To assess how the primary and secondary outcome measures change over time, we will fit linear mixed models to each outcome with the primary independent variable in each model being treatment site. The models will include covariates including participant characteristics, measures of social support, and exploratory measure of hair cortisol. Interactions between time and model will be included in each mixed model to determine whether measures change differentially between the sites over time. To address potential heterogeneity of treatment effects for poverty status (less than FPL versus between FPL and 250% FPL), we will also include an interaction term between poverty status and site. We will report adjusted least-squares mean difference estimates and appropriate confidence intervals of the outcome measures.

#### Qualitative analysis

We will complement our quantitative analyses by conducting analyses of data from key staff/provider interviews (Aim #1), and patient/social support interviews and focus groups (Aims #1 & 2). We will follow criteria for a theory-driven qualitative content analysis [43]. We will identify themes, and code and subcode transcripts to explore interconnections between theme categories and to develop a holistic interpretation of the data.

## Discussion

It is common in studies of diabetes to have the primary outcome be A1c levels. But in this patient-engaged study, patients wanted us to take a different approach. We know how to get people to lower their A1c; the issue is getting them to do those things. Patients told us that you have to understand your diabetes, have the capacity to make changes in your life, and know how to control your stress before you can even think about lowering your A1c. Therefore, we said that “capacity” is measured by our two primary outcomes (diabetes knowledge and diabetes-related patient activation) and the success of the capacity is measured by the biological measures of A1c, BMI, depression, and cortisol. We hypothesize that the program that is most culturally and contextually situated will do the best job educating people about their diabetes in a meaningful, lasting way, helping them to become empowered (activated), and helping them or giving them strategies to deal with stress in their lives. Therefore the most culturally and contextually situated program will be the most “culturally competent” and will have the best impact on capacity for diabetes self-management (as measured by diabetes knowledge and patient activation in relation to diabetes health). We also expect to see primary, secondary, and exploratory outcomes to be correlated--e.g.*,* patients with better knowledge and activation are likely to have lower A1c levels, lower BMI, improved depression scores, and lowered cortisol levels.

In addition, patients told us that the common approach to measuring diabetes control at the individual patient level fails to appreciate the cultural and social context of people’s lives, which they believe is essential for understanding diabetes self-management for Latinos. To attempt to capture this social dimension of people’s lives, we are recruiting patients and social supports as dyads. Patient outcomes will be measured and information we gather from social supports will inform our understanding of those results.

Obstacles we anticipate include confounders that may influence statistical analyses, attrition in participation that may make data collection difficult, and identification of A1c and depression levels that could require that we refer patients for further care. At the end of this 3-year study we expect to be able to identify the characteristics of diabetes self-management programs that make them culturally competent.

## Abbreviations

A1c: HbA1c blood test for glycated hemoglobin or glycosylated hemoglobin to measure blood sugar level
BMI: Body mass index
CAHPS-CC: The Consumer Assessment of Healthcare Providers and Systems Cultural Competence Set
CCM: The chronic care model
DKQ: The diabetes knowledge questionnaire
DSMS: The Diabetes Self-Management Support Empowerment Model (DSMS)
FPL: Federal poverty level
PAM-13: The patient activation measure (PAM-13
PCP: Primary care provider
pg/mg: Picogram of cortisol per milligram of hair
PHQ-9: The patient health questionnaire 9
REDCap: Research Electronic Data Capture - a browser-based clinical and translational research database software program
